# Index to the Transactions of the Epidemiological Society of London

**Published:** 1858-04

**Authors:** 


### INDEX.

Army, French, Dr. Milroy on sickness and mortality in, 21
Babington, Dr. B. G., introductory address, 73
Barker, Dr. T. H., origin and propagation of certain epidemic diseases, 79
Cholera, Mr. F. H. Johnson on localised causes of, 53
Diseases, Mr. Michael on difficulties in study of, 65
Epidemic diseases, Dr. Barker on origin and propagation of, 79
Epidemiological Society, annual report of Council, 44; quarterly reports, of proceedings, 20, 72
Fever on board a Turkish line of battle ship, Mr. Radcliffe on, 13
Introductory Address, 1858-9, 73
Johnson, Mr. F. H., localised causes of cholera, 53
Michael, Mr. W. H., difficulties of the study of prevailing diseases, 65
Milroy, Dr. G., sickness and mortality in the French army, 21
Radcliffe, Mr. J. N., fever on board the Turkish line of battle ship Tesherefieh, 13
Seaton, Dr. E. C., small-pox in Jamaica in 1851-2, 1
Small-pox in Jamaica, Dr. Seaton on, 1

